# Supplementation with eicosapentaenoic and docosahexaenoic acids during late gestation alters fatty acid profiles in ewe colostrum, milk, and plasma, and lamb plasma

**DOI:** 10.1093/jas/skaf366

**Published:** 2025-11-16

**Authors:** Ana Cristina Carranza-Martin, Kirsten Nickles, Danielle Nicole Sherlock, Alejandro E Relling

**Affiliations:** UCA, CONICET, Animal Reproduction Laboratory, Buenos Aires, Argentina; Department of Animal Science, The Ohio State University, Wooster, OH 44691; Department of Animal Science, The Ohio State University, Wooster, OH 44691

**Keywords:** colostrum, plasma, Polyunsaturated fatty acids, sheep

## Abstract

Our aim was to evaluate the effects of increasing dietary levels of eicosapentaenoic acid (EPA) and docosahexaenoic acid (DHA) during the final third of gestation on plasma and milk fatty acid (FA) profiles of ewes and their offspring. Additionally, correlation between maternal and offspring plasma, colostrum, and milk FA profiles were examined. Seventy-two pregnant ewes (92.2 ± 2.94 kg body weight at d 100 of gestation) were blocked by body condition score and randomly assigned to one of three diets containing 0, 1, or 2% of a Ca salt of FA enriched with EPA and DHA during the last 50 d of gestation. Animals were housed in 8 pens per treatment (3 ewes per pen) during the supplementation period. At lambing, all ewes were penned together and offered a diet without FA supplementation. For FA profile, blood samples were collected from ewes at d -20 prepartum, at lambing, and d 15 postpartum. Lamb plasma samples were obtained at birth (0.5 to 8 h post-suckling). Colostrum (0.5 to 8 h postpartum) and milk (d 15 postpartum) were also collected. Data were analyzed using a randomized complete block design. The model included the fixed effects of the treatment for lamb plasma FA profile; and the fixed effects of treatment, time, and their interaction for ewe plasma and colostrum and milk FAprofile. Supplementation with EPA and DHA increased (diet by time interaction; *P *≤ 0.06) the polyunsaturated FA (PUFA; C20:40, C20:5, and all omega 3) in ewe plasma until lambing but was similar between treatments 15 d post lambing. Colostrum from supplemented ewes showed greater PUFA (C22:5 and C22:6) concentrations and the difference decreased in the milk (diet by time interaction; *P *< 0.01), although the colostrum PUFA increase was not correlated with most individual plasma PUFA (C18:2, C20:3n6 and n3, C20:4, C20:5, C22:5, and C22:6), except for C18:3, which showed a positive association (*P *= 0.03). Furthermore, colostrum PUFA concentrations were positively correlated with the FA composition of lamb plasma post-suckling (*P *< 0.02). The PUFA concentrations were also increased in lamb plasma at lambing (*P *< 0.01), suggesting potential effects on neonatal lipid metabolism. These findings demonstrate that dietary supplementation with EPA and DHA, initiated 50 days before lambing and terminated at parturition, can alter the FA composition of maternal plasma, colostrum, and milk, and also influence the plasma FA profile of the offspring.

## Teaser

This experiment shows that supplementing ewes with eicosapentaenoic acid (EPA) and docosahexaenoic acid (DHA) in late gestation can boost the nutritional quality of colostrum and increase the concentration of these fatty acids in the lamb.

## Introduction

Feeding ruminants with different types of fatty acids modifies the fatty acid composition of their tissues, colostrum, and milk (Shingfield et al., 2013). Furthermore, omega-3 (n-3) fatty acids (n3LCFA), eicosapentaenoic acid (EPA), and docosahexaenoic acid (DHA), act as bioactive molecules, which can activate transcription factors (Carranza-Martin et al., 2025). These n3LCFA can enhance the transcription of lipolytic genes while reducing the transcription of lipogenic genes (Clarke, 2001). Previously, supplementing ewes with a lipid source containing EPA and DHA during late gestation alters the relative mRNA abundance of subcutaneous adipose tissue genes linked to lipid metabolism (Coleman et al., 2018a; Nickles et al., 2019). Furthermore, maternal supplementation with a lipid source containing EPA and DHA during late gestation increased offspring body weight during finishing stage (Nickles et al., 2019). Therefore, increasing EPA and DHA concentration in both ewes and lambs could positively affect their health and metabolism.

Despite n3LCFA often being grouped together as having similar biological functions, there are notable differences in their specific roles; for example, DHA is preferentially incorporated into phospholipids; whereas EPA is more commonly incorporated into cholesterol esters (Offer et al., 2001; Urrutia et al., 2023). However, the incorporation of DHA and EPA into triacylglycerols or non-esterified fatty acids is minimal (Zymon et al., 2014). Due to this incorporation, there is a limited uptake of these fatty acids by the mammary gland, mainly because the mammary gland obtains most of its fatty acids from lipoproteins (Moore and Christie, 1979). However, it was observed that supplementation with an enriched source of EPA and DHA during the last 50 d of gestation modified the fatty acid profiles of colostrum, plasma of ewes and lambs, and milk at 30 d postpartum (Coleman et al., 2018a).

Changes observed in the lipid profile of plasma and milk 30 d postpartum following EPA and DHA supplementation during late gestation raises questions about what occurs earlier in the postpartum period. Furthermore, studies have focused on comparisons between long-chain monounsaturated and polyunsaturated fatty acids or multiple combinations of long chain fatty acids sources (Dunstan et al., 2007; Moallem and Zachut, 2012; Gallardo et al., 2014; García et al., 2014; Elis et al., 2016; Coleman et al., 2018a), but little is known about the effects of increasing concentrations of polyunsaturated fatty acids in comparison with unsupplemented animals. We hypothesized that increasing concentrations of EPA and DHA during late gestation increases their concentration in the ewe’s plasma, colostrum, milk, and in the offspring’s plasma. Additionally, we hypothesize a positive correlation between the ewe’s plasma fatty acid profile and those in colostrum and milk, as well as a positive correlation between the fatty acid profiles in colostrum and milk and the lamb’s plasma. The objective of this Experiment is to assess the impact of increasing doses of EPA and DHA in the diet of ewes during the last third of gestation on the peripartum ewe′s plasma and milk fatty acid profiles, and on the plasma fatty acid profile of their offspring. Furthermore, we aim to evaluate the association between plasma (maternal and offspring) and colostrum and milk fatty acid profiles.

## Materials and Methods

This experiment was conducted at the Sheep Research Center of the Ohio Agricultural Research and Development Center, Wooster Campus, The Ohio State University OH (IACUC #2016A00000013).

### Experimental design and sampling

The procedure of this experiment was explained previously by Nickles et al. (2019). Briefly, seventy-two pregnant ewes with an initial body weight of 92.2 ± 2.94 kg at d 100 of gestation were blocked by body condition score (BCS), and within each block were randomly assigned to one of the three different treatments. Groups of 24 ewes (8 pens per treatment and 3 ewes per pen) were fed one of three diets containing 0%, 1%, or 2% of a calcium salt of fatty acids enriched with EPA and DHA (Strata G113, Virtus Nutrition LLC, Corcoran, CA) based on the DMI. Ewes received 2.02 kg/d of a mixed diet containing corn silage (30.54%), alfalfa haylage (17.96), and a concentrate (51.49%), which contained the different treatments (**Table 1**). The fatty acid composition of the diets is in **Table 2**. The concentrate contained dry distiller’s grains, soybean hulls, mineral and vitamin premix, and fatty acid. As the fatty acid supplement increased in the diet, there was a decrease in dry rolled corn. The feed intake was fixed to meet, but not exceed, the nutrient requirements for ewes in late gestation (NRC, 2007). At lambing, the dietary treatments were discontinued, and all ewes and lambs were moved to a common pen, where they received the same diet. In previous studies evaluating fatty acid (FA) supplementation during late gestation (Garcia et al., 2014; Marques et al., 2017; Carranza Martin et al., 2018; Coleman et al., 2018a, 2018b, 2019) or across late gestation and early lactation (Palmquist et al., 1977; Reynolds et al., 2002; Capper et al., 2007), diets typically contained comparable amounts of total lipid but differed in their FA profiles. The rationale for altering lipid sources rather than increasing the overall lipid content lies in the fact that a higher FA concentration could confound the interpretation of results by altering dietary energy supply. In the present experiment, ewes supplemented with 1 or 2% EPA+DHA consumed diets with greater net energy for maintenance (NEm; 0.54 and 1.07%, respectively) compared with unsupplemented ewes (0% EPA+DHA; FEDNA, 2010). The diets were not isoenergetic, since achieving this would have required the inclusion of alternative ingredients such as corn, dried distillers grains, or fiber. However, as Radunz et al. (2011) demonstrated, offspring growth and metabolism can be influenced when dams are fed isoenergetic diets formulated with different feedstuffs. Thus, adding new feed ingredients to equalize energy in the current experiment could have confounded the effects of FA supplementation with those arising from other dietary components. Although the diets were not isoenergetic, the increase in NEm intake of 0.54% per treatment is unlikely to fully explain the differences observed in this experiment.

**Table 1.** Diet ingredients and composition (% of DM basis) of diets containing 0, 1, or 2% of supplemented enriched sources of EPA and DHA, fed to pregnant ewes at 2 kg/d during the last 50 d of gestation.

	% of DM basis		
	0 %	1 %	2 %
**Ingredients**			
** Corn silage**	30.54	30.54	30.54
** Alfalfa haylage**	17.96	17.96	17.96
** Ground corn**	10.10	8.89	7.97
** Soybean hulls**	30.65	30.88	30.81
** limestone**	0.44	0.48	0.48
** DDGS^1^**	10.10	10.07	10.04
** Mineral supplement^2^**	0.20	0.18	0.18
** Fatty acid supplement^3^**	—	1.01	2.03
**Composition**			
** Crude Protein**	15.48	14.41	14.47
** NDF^4^**	39.21	41.27	41.63
** EE^4^**	2.21	2.96	3.63
** Ash**	5.18	5.38	5.68

1 DDGS = Distiller’s dried grains with solubles.

2 Vitaferm Concept-Aid Sheep (BioZyme, St Joeseph, MO). Contains 15.5% Ca, 5% P, 16% NaCl, 4% Mg, 2% K, 10 ppm Co, 70 ppm I, 2850 ppm Mn, 16.4 ppm Se, 2500 ppm Zn, 130000 IU/kg vitamin A, 7500 IU/kg vitamin D_3_, 550 IU/kg vitamin E.

3 Strata G113, Virtus Nutrition LLC, Corcoran, Ca.

4 NDF: Neutral detergent fiber; EE: Ether extract.

**Table 2.** Fatty acid profile (% of total FA) of the fat supplement fed to pregnant ewes during the last 50 days of gestation.

Fatty Acid	Supplement^1^
**C12:0**	0.12
**C14:0**	3.95
**C16:0**	21.99
**C16:1**	7.40
**C18:0**	7.46
**C18:1**	17.44
**C18:2**	2.69
**C20:0**	0.34
**C18:3**	0.92
**C20:5**	9.18
**C22:6**	6.99
**Other**	22.00

1 StrataG113 as a source of EPA and DHA (Virtus Nutrition LLC, Corcoran, Ca).

Blood samples from the ewes were collected on d -20 prepartum, at lambing, and d 15 postpartum. Blood samples from lambs were collected on the d of birth after colostrum (0.5 to 8 hours post suckling) and on d 15; however, the d 15 samples lost their cold chain and could not be used for analysis. Samples of 10 and 5 mL were taken from the jugular vein of ewes and lambs, respectively, immediately transferred into polypropylene tubes (Sarstedt, Nümbrecht, Germany) containing solutions of disodium EDTA and benzamidine HCL (1.6 mg and 4.7 mg/mL of blood, respectively), and placed on ice. After centrifugation for 25 min (1,800 × g at 4 °C), plasma was aliquoted and stored at −80°C until fatty acid analysis. Colostrum samples were taken from ewes at lambing (0.5 to 8 hours after parturition) and stored in 10 mL polypropylene tubes on ice until the fat was separated by centrifugation at 20,000 × g for 30 min at 4 °C. Following centrifugation, the fat layer was removed and stored at −80°C until fatty acid analysis (Coleman et al., 2018a). Milk samples (d 15 postpartum) were collected from each ewe manually and treated with bronopol and natamycin (Advanced Instruments, Norwood, MA) as preservatives and stored in polypropylene tubes placed on ice until centrifugation for fat separation as described above for colostrum.

### Fatty acid analysis

The fatty acid profile of both colostrum and milk were determined using a two-step procedure for methylation (Jenkins, 2000). Total lipids in plasma were extracted as described previously by Folch et al. (1957) with modifications made by Coleman et al. (2018a). Briefly, 20 mL of a 2:1 chloroform–methanol ratio (vol/vol) was added to 1 or 0.5 mL of plasma for ewes and lambs, respectively. Samples were then vortexed and left to stand for 5 min before being filtered through a Whatman No. 1 filter paper (GE Healthcare Bio-Sciences, Pittsburgh, PA). Distilled H_2_O was added (4 mL) and then samples were vortexed and centrifuged at 3,000 rpm for 4 min. The upper phase was discarded, and the interface was then rinsed three times with 3 mL of a 3:48:47 chloroform: methanol: water mixture (vol/vol/vol) and discarded. Samples were evaporated under N_2_, and lipids were recovered in 1 mL of hexane. Extracted fatty acids were then methylated as described previously by Doreau et al. (2007). The hexane layer was placed in a gas chromatography vial and stored at −20°C until analysis. All fatty acid methyl esters were separated by gas chromatography using a CP-SIL88 capillary column (100-m × 0.25-mm × 0.2-μm film thickness; Varian Inc., Palo Alto, C). The Injector and flame ionization detector temperatures were set as described previously (Reveneau et al., 2005). Retention times were determined with the standards GLC60 and 68D from NuChek Prep (Elysian, MN) and 47080U from Supelco Inc. (Bellefonte, PA).

### Statistical analysis

All data were analyzed as a randomized complete block design with repeated measures when needed, using the MIXED procedure of SAS (SAS Institute, Cary, NC) with a model testing the fixed effects on treatment, time (d of the sampling), and their interaction (for ewe plasma FA and colostrum and milk concentrations; lamb plasma FA concentration did not include time and the interaction). The model included the random effects of block and pen within block. Pen was considered as the experimental unit, and time was included as a repeated measure when needed. For the analysis with repeated measurements, different covariate structures were compared (unstructured, autoregressive, compound symmetry, and variance components); the compound symmetry structure was used based on the lowest Akaike Information Criterion. If there was no interaction between treatment and time (*P* > 0.10), orthogonal polynomial effects, linear and quadratic, were evaluated. Least square means (LSMEANS) and standard errors of the mean (SEM) were determined using the LSMEANS statement in each procedure. Two Pearson correlation procedures were conducted. The first one was between the concentration of fatty acids in colostrum and the concentration of fatty acids in lamb plasma, while the second was between the concentration of fatty acids in colostrum and fatty acid concentration in ewes’ plasma at lambing day. Differences were set at *P* ≤ 0.05, and marginal differences were determined at *P* > 0.05 and *P* ≤ 0.10.

## Results and Discussion

To facilitate the readability of the results section, the results are presented first as the differences due to the interaction of treatment and time, then we present the results of the difference due to treatment (as linear and quadratic effects), finishing presenting the data of the ones with no difference. For that reason, the tables with the differences are split following the same order.

### Ewe plasma fatty acid profile

In line with other studies in sheep, the main fatty acids in plasma measures from all treatments were C16:0, C18:0, C18:1c9, and C18:2 (Capper et al., 2007; Or-Rashid et al., 2010; Coleman et al., 2018a). In our experiment, a treatment by time interaction was observed in C15:0 (P < 0.01; Table 3) where both the 0 and 1% supplementation resulted in a decrease in concentration, while 2% supplementation led to increased concentration throughout the experiment. Conversely, C16:1 and C17:0 also exhibited a treatment by time interaction (P < 0.01), with concentrations increasing in the 0% group over time, while supplemented groups (1% and 2%) showed lower concentrations. The fatty acid C16:0 also exhibited a treatment by time interaction (P < 0.01). On d -20, plasma concentration of C16:0 was greatest in 2% supplemented ewes. However, by d 15 the concentration of C16:0 had decreased across all treatments, and the control group (0% supplementation) displayed a greater concentration than the 2% group. Previously, a treatment by time interaction was observed for C16:0 when lactating ewes were supplemented for 42 d with a mix of soybean and fish oil compared to ewes that did not receive oil supplementation; non-supplemented ewes had greater C16:0 concentration than supplemented ones, and this concentration decreased over time (Tsiplakou and Zervas, 2013). A treatment by time interaction was observed for C18:0 (P < 0.01). The 0% ewes presented a greater concentration than 1% and 2% initially, but over time, C18:0 decreased in the 0% group, while it increased in the supplemented groups, which is consistent with findings by Coleman et al. (2018). In that experiment, on d 20 prepartum, ewes supplemented with calcium salts of a palmitic fatty acid distillate had greater plasma concentrations of C18:0 than those supplemented with EPA + DHA. The observed reductions in C18:0 concentration during parturition could be related to colostrum production; C18:0 present in colostrum and milk originates in the bloodstream, suggesting greater absorption by the mammary gland and lower circulating concentration on the day of parturition (Enjalbert et al., 1998). Furthermore, this decrease could also be due to a dilution effect resulting from the greater mobilization of fatty acids from adipose tissue during parturition to meet the higher energy demands of milk production (Drackley, 1999).

**Table 3.** Interaction effect of increasing concentration of EPA and DHA supplementation (0%, 1%, 2% of calcium salts containing EPA and DHA) to ewes during the last 50 d of gestation on ewe plasma fatty acid profile before parturition (d -20), the day of lambing (d 0), and d 15 after parturition (% of total fatty acid methyl esters).

	Treatment (Trt)				SEM	*P*-Values		
Fatty acid	Day	0%	1%	2%		Trt	Day	Trt × day
**C15:0 ante**	−20	0.47	0.32	0.13	0.06	0.23	<0.01	<0.01
	0	0.15	0.02	0.06	0.07			
	15	0.13	0.22	0.22	0.06			
**C16:0**	−20	17.10	19.60	20.83	0.91	0.28	<0.01	<0.01
	0	13.97	12.87	13.46	0.99			
	15	16.28	15.39	13.46	0.93			
**C16:1 & C17:0 ante**	−20	0.79	1.34	1.76	0.13	0.04	0.05	<0.01
	0	1.18	0.98	1.18	0.14			
	15	1.19	1.22	1.11	0.14			
**C18:0**	−20	21.23	18.59	14.92	0.97	0.02	<0.01	<0.01
	0	17.82	12.86	15.27	1.00			
	15	19.60	19.86	19.79	0.99			
**C18:1t6**	−20	0.23	0.49	0.35	0.07	0.65	<0.01	0.03
	0	0.22	0.20	0.11	0.08			
	15	0.20	0.16	0.25	0.07			
**C18:1t10**	−20	1.15	2.30	3.33	.019	<0.01	<0.01	<0.01
	0	0.54	0.70	0.80	0.21			
	15	0.26	0.44	0.50	0.19			
**C18:1t11**	−20	1.89	3.66	5.23	0.19	<0.01	<0.01	<0.01
	0	1.00	1.63	2.09	0.21			
	15	0.87	1.00	1.14	0.19			
**C18:2t10c12**	−20	0.14	0.27	0.06	0.06	0.13	<0.01	0.10
	0	0.39	0.25	0.21	0.07			
	15	0.16	0.11	0.15	0.06			
**C22:0**	−20	0.11	0.04	0.00	0.04	0.41	<0.01	0.03
	0	0.20	0.08	0.05	0.05			
	15	0.02	0.06	0.00	0.04			
**C22:1**	−20	4.47	2.77	2.91	0.28	<0.01	<0.01	0.03
	0	1.40	1.03	1.06	0.32			
	15	1.45	1.48	0.93	0.30			
**C20:4**	−20	0.03	0.00	0.00	0.20	<0.01	<0.01	0.06
	0	1.81	0.88	0.93	0.23			
	15	1.17	0.50	0.23	0.21			
**C20:5**	−20	0.86	1.38	3.93	0.19	<0.01	<0.01	<0.01
	0	0.44	0.78	2.13	0.22			
	15	0.65	0.78	1.14	0.20			
**C22:5**	−20	1.98	2.23	2.09	1.65	0.36	<0.01	0.06
	0	1.50	8.46	7.38	1.83			
	15	4.81	5.30	3.85	1.69			
**n3**	−20	6.07	7.45	10.54	1.69	0.04	0.48	0.02
	0	3.79	11.40	13.05	1.86			
	15	8.12	9.05	8.59	1.72			
**EPA + DHA**	−20	2.07	3.39	6.97	0.33	<0.01	<0.01	<0.01
	0	1.18	2.27	4.82	0.38			
	15	1.16	1.79	2.73	0.35			

Polyunsaturated fatty acids are targets of ruminal biohydrogenation, resulting in the formation of trans isomers (Bauman and Griinari, 2003). Therefore, changes in the C18:1 isomers are indicative of changes in biohydrogenation with EPA and DHA supplementation. In our results, we observed that C18:1t6, C18:1t10, and C18:1t11 showed treatment by time interactions (*P *≤ 0.03) and a marginal interaction effect for C18:2t10c12 (*P *= 0.10). The C18:1t6 concentration was greater with 1% supplementation but decreased over time for all treatments, ending with the 1% group showing the lowest concentration (Table 3). Both C18:1t10 and C18:1t11 decreased by d 15 postpartum in all treatments but supplemented animals had greater concentrations as DHA and EPA in the diet increased. Our results are consistent with Coleman et al. (2018), where at d 20 prepartum (after 30 d of supplementation) control ewes (supplemented with calcium salts of a palmitic fatty acid distillate) presented with lower concentrations of C18:1t10 and C18:1t11 than EPA + DHA supplemented ewes; and by d 30 postpartum, the values of this fatty acids decreased for all treatments. In the present trial, C18:2t10c12 exhibited greater concentration in the 1% diet group compared to the control (0%) and 2% groups on d -20. However, by d 0, C18:2t10c12 increased in the 0% and 2% groups while remaining unchanged in the 1% group. By d 15, the concentration had decreased across all diets. Our results contrast with previous studies that found no differences in C18:2t10c12 between control and supplemented ewes (Tsiplakou and Zervas, 2013; Coleman et al., 2018a). This discrepancy may be attributed to differences in the timing of supplementation; in our experiment, ewes were not supplemented after parturition, whereas in Tsiplakou and Zervas (2013), supplementation was throughout lactation. Additionally, differences in supplementation concentration could be a factor, as Tsiplakou and Zervas (2013) used a single dose of a blend of fish oil and soybean oil. Furthermore, in the experiment by Coleman et al. (2018), the control group received supplementation with calcium salts of a palmitic fatty acid distillate, which may have influenced their results compared to the current experiment.

A treatment by time interaction was observed for C22:0, C22:1, C20:5, total n-3, and EPA + DHA (P < 0.03; Table 3), while a marginal difference for an interaction was observed for C20:4 and C22:5 (*P *= 0.06). At d -20, the 0% group showed a greater concentration of C22:0 than supplemented ewes. By d 0, C22:0 concentration increased across all treatments. Subsequently, by d 15 postpartum, C22:0 concentrations decreased again in all groups. The plasma concentration of C22:1 also showed a greater concentration in the 0% group than the supplemented groups at d -20 prepartum; then it decreased sharply on d 0 for all treatments and did not change by d 15 postpartum. Plasma concentrations of C20:4 increased at d 0 and decreased again by d 15 postpartum for all treatments, but the magnitude of increase at d 0 was greatest in the 0% group. The plasma concentration of C20:5 was greater at d -20 prepartum as the dose of EPA and DHA increased; by d 0 the concentration of C20:5 decreased for all treatments, but 0% and 1% had similar concentration, while ewes in 2% had a greater concentration. On d 15 postpartum, C20:5 remained similar for 0% and 1%, but it decreased in the 2% supplemented ewes. However, the concentration of C20:5 was still greater on day 15 postpartum in 2% compared with the 0% and 1% treatment. The concentrations of C22:5 and total n-3 were greater in the supplemented animals compared to the control, concentrations increasing from d -20 prepartum until d 0 for the 1% and 2% compared with the control, but then decreasing after parturition; all three treatments had similar concentrations at d 15 postpartum.

In a previous experiment (Coleman et al., 2018a), a treatment by day interaction was observed for DHA in ewes supplemented with EPA + DHA, with greater plasma concentrations at the beginning of supplementation, which decreased over time, similar to our results. Our supplementation of EPA + DHA is reflected in the greater total EPA and DHA content, and the total n-3 content in the plasma of ewes compared to those without supplementation. The increase in EPA and DHA plasma concentration also aligns with other studies in which ruminants were fed fish meal (Or-Rashid et al., 2012) or fish oil sources (Capper et al., 2007; Elis et al., 2016). In all these experiments, plasma concentrations of EPA and DHA are aligned with the presence of these two fatty acids in the diet. Although supplementation ended on d 0 in the present experiment, it is possible that lipid regulatory mechanisms remained active beyond the duration of the diet.

Additionally, there was a quadratic effect for plasma C18:1c15 (*P *= 0.01; Table 4) and a marginal quadratic effect for C17:1 (*P *= 0.07), where 1% supplementation increased their concentration compared with 0% and 2% groups. A linear effect, where the control group had a greater concentration than the supplemented ewes, was observed for C18:1c9 and C18:1c13 (*P *< 0.03), while the same marginal linear effect was observed for C18:2t10c12 (*P *= 0.06). The C18:1c9 has been reported to be lower in lactating ewes without oilseed supplementation compared with those supplemented with oilseeds (canola, sunflower, or flaxseed) for 21 days (Zhang et al., 2006). Our results are consistent with these findings, as the control group in our experiment did not receive fatty acid supplementation. Similarly, C18:2t10c12 showed no differences in plasma from ewes supplemented with palmitic and oleic acid compared with DHA + EPA supplementation (Coleman et al., 2018a). In our experiment, the absence of fatty acid supplementation may have altered the distribution of this fatty acid, resulting in higher levels in control animals. On the other hand, C22:6 (*P *< 0.01), and C20:3n6 (*P *= 0.06) showed a linear and marginal linear increase, respectively, as the supplementation rate of EPA and DHA increased. Previously, in lactating ewes, ewes supplemented with fish oil and soybeans had greater plasma concentrations of C22:6n-3 than the control (Tsiplakou and Zervas, 2013). Also, cows supplemented with flaxseed oil or fish oil from d 256 of gestation to calving presented with greater concentrations of C22:6n-3, C22:5n-3, and total n3LCFA in plasma compared to those ones supplemented saturated fatty acids (Moallem and Zachut, 2012).

**Table 4.** Linear or quadratic effects of increasing concentration of EPA and DHA supplementation (0%, 1%, 2% of calcium salts containing EPA and DHA) to ewes during the last 50 d of gestation on the ewe plasma fatty acid profile at d -20 before parturition, the day of lambing (d 0), and d 15 after parturition (% of total fatty acid methyl esters).

	Treatment (Trt)				SEM	*P*-Value		
Fatty acid	Day	0%	1%	2%		Linear	Quadratic	Trt × day
**C17:1**	−20	0.41	0.34	0.07	0.18	0.18	0.07	0.62
	0	0.37	0.79	0.17	0.21			
	15	0.34	0.39	0.23	0.19			
**C18:1c9**	−20	16.09	12.23	9.28	1.16	<0.01	0.65	0.19
	0	14.68	11.32	11.59	1.22			
	15	15.38	15.13	12.76	1.22			
**C18:1c13**	−20	0.09	0.15	0.09	0.05	0.03	0.75	0.23
	0	0.34	0.17	0.13	0.06			
	15	0.43	0.36	0.35	0.05			
**C18:1c15**	−20	0.01	0.03	0.00	0.02	0.71	0.01	0.33
	0	0.03	0.03	0.02	0.02			
	15	0.06	0.15	0.07	0.02			
**C20:3n6**	−20	0.35	0.78	0.80	0.10	0.06	0.41	0.14
	0	0.20	0.17	0.20	0.12			
	15	0.24	0.30	0.33	0.11			
**C22:6**	−20	1.23	2.02	3.02	0.20	<0.01	0.44	0.25
	0	0.75	1.49	2.69	0.23			
	15	0.52	1.04	1.59	0.21			

Finally, those fatty acids that did not show significant effects (*P *≥ 0.14) are shown on Supplementary Table 1. Surprisingly, and contrary to expectations, no significant differences were observed between supplemented and control ewes in the concentrations of C20:3n-3 and n-6 fatty acids. Previous reports described an increase in C20:3n-3 in the plasma of ewes supplemented with EPA + DHA on d 30 postpartum/after supplementation ended (Coleman et al., 2018a), as well as a time-dependent increase in C20:3n-3 in ewes supplemented with soybean and fish oil (Tsiplakou and Zervas, 2013). Additionally, we expected to find greater n-6 concentration in plasma of supplemented ewes, as previously reported with enhanced dietary EPA and DHA (Tsiplakou and Zervas, 2013; Coleman et al., 2018a). The lack of differences in both fatty acids, C20:3n-3 and n-6, may be attributed to variations in control groups, supplementation duration, and concentrations used. Nonetheless, further research is needed to clarify these findings.

### Colostrum and milk fatty acid profile

Fatty acids C16iso, C18:1t6/8, C18:1t10, C18:1t11, C18:1c11, C18:1c13, C18:1c15, C18:1c16, C22:0, C18:2, C18:2c9t11, C20:3n6, C20:4, C20:5, C22:0, C22:1, C22:5, and C22:6 exhibited a treatment by time interaction effect (*P *≤ 0.04; Table 5). For C16iso, concentrations were initially lower in the colostrum of supplemented ewes compared to the 0% group, but it increased over time, becoming greater in the milk of supplemented ewes than control (0%) ewes. The fatty acids C18:1c13 (*P *< 0.01) and C18:1c15 (*P *< 0.01) increased in the 0% and 2% groups from colostrum to milk, while they remained unchanged in the 1% group. Palmitoleic acid (C18:1c16) increased from colostrum to milk in supplemented ewes, but the 0% group showed a decrease over time (*P *< 0.01). Concentrations of C22:0 were similar across treatments in colostrum but were greater in milk of supplemented ewes, especially in the 1% group (*P *< 0.01). Conversely, C22:1 increased from colostrum to milk in the 0% group but did not change in supplemented treatments (*P *< 0.01). Notably, C18:1t10, C18:1t11, C18:1c11, C18:2c9t11, C20:3n6, C22:5, and C22:6 were all greater in both colostrum and milk of supplemented ewes than 0% ewes, with a decrease in their concentrations over time (*P *< 0.01). The C18:2 concentration was greater in the colostrum of the 0% ewes but lesser in milk compared to supplemented groups (*P *< 0.01). Arachidonic acid (C20:4) was lower in the 1% group in colostrum compared to the 0% and 2% groups. However, its concentration remained unchanged in milk for the 1% group, while it decreased in the 0% and 2% groups, resulting in greater concentration in the 1% ewes’ milk (*P *< 0.01). The 2% group had the greatest concentration of C25:5 in colostrum but the lowest in milk, with no changes observed for the 0% and 1% groups (*P *< 0.01). Lastly, C18:1t6/8 showed a marginal interaction (*P *= 0.07, Table 5), with a decrease in the 1% group from colostrum to milk, while the 0% and 2% groups showed slight increases. The fatty acid C18:1t11 could reduce milk fat fluidity due to its higher melting point, prompting adaptations in the mammary gland to maintain fat fluidity (Gallardo et al., 2014). It has been suggested that de novo fatty acid synthesis may increase to counter the rise in C18:1t11 concentration, ensuring fluidity in milk fat (Gallardo et al., 2014). The formation of this isomer is linked to changes in biohydrogenation pathways. High concentration of this isomer in colostrum of supplemented ewes suggests that biohydrogenation pathways were altered by fatty acid supplementation (Bauman and Griinari, 2003). Previously, a treatment by time interaction was observed for several C18:1 isomers: trans-10, cis-12, cis-15, cis-9, trans-11, and C18:2 (Coleman et al., 2018a). However, these interactions were different from our results. In both Coleman et al. (2018a) and the current experiment, the concentration of C18:1t10 was greater in colostrum. However, in our milk results, the concentration of this fatty acid decreased in all treatments equally but, in supplemented ewes, the concentration was greater. This did not happen in Coleman et al. (2018), where all treatments had the same concentration in milk. Additionally, in both studies, Coleman et al. (2018) and the current research, there was a treatment by day interaction for C18:2c9t11 with greater concentration in colostrum of ewes supplemented with EPA + DHA. But in Coleman et al. (2018), in milk, the concentration in the control group (calcium salts of a palmitic fatty acid distillate) increased, and in the current experiment the concentration of C18:2c9t11 decreased in all treatments. These differences between the studies could be due to differences in the control group (no fat supplementation vs. calcium salts of a palmitic fatty acid distillate), or the time of the milk samples between the two studies (15 vs 30 d postpartum). Finally, the increased levels of C20:4, C20:5, C22:5, and C22:6 in colostrum, particularly in ewes fed with DHA and EPA, reflect the different requirements depending on the physiological stage for these fatty acids. Similar increases in colostrum have been observed in primiparous and multiparous cows (Wilms et al., 2022), suggesting a preferential uptake by the mammary gland to meet the neonate’s needs for these fatty acids.

**Table 5.** Interaction effects on the fatty acid profile of colostrum (d 0) and milk at 15 d in lactation of increasing concentration of EPA and DHA supplementation (0%, 1%, 2% of calcium salts containing EPA and DHA) to ewes during the last 50 d of gestation (% of total fatty acid methyl esters).

	Treatment (Trt)				SEM	*P*-Value		
Fatty acid	Day	0%	1%	2%		Trt	Day	Trt × day
**C16 iso**	0	0.18	0.15	0.15	0.01	0.27	<0.01	<0.01
	15	0.23	0.30	0.30	0.02			
**C18:1 t6/8**	0	0.34	0.38	0.30	0.02	0.37	0.51	0.07
	15	0.35	0.29	0.32	0.03			
**C18:1 t10**	0	1.26	2.08	3.13	0.22	<0.01	<0.01	<0.01
	15	0.70	1.38	1.22	0.27			
**C18:1 t11**	0	1.13	1.72	2.34	0.17	<0.01	<0.01	0.04
	15	1.21	1.40	1.52	0.22			
**C18:1 c11**	0	0.61	0.71	0.94	0.05	<0.01	<0.01	<0.01
	15	0.36	0.65	0.53	0.06			
**C18:1 c13**	0	0.04	0.05	0.05	0.02	0.05	<0.01	<0.01
	15	0.19	0.06	0.13	0.02			
**C18:1 c15**	0	0.07	0.06	0.07	0.01	<0.01	<0.01	0.01
	15	0.18	0.07	0.12	0.02			
**C18:1 c16**	0	0.13	0.14	0.10	0.02	<0.01	<0.01	<0.01
	15	0.12	0.29	0.19	0.03			
**C18:2**	0	3.36	2.76	2.75	0.15	0.31	<0.01	<0.01
	15	2.51	2.78	2.71	0.18			
**C18:2 c9t11**	0	1.02	1.48	1.77	0.10	<0.01	<0.01	0.01
	15	0.57	0.68	0.69	0.13			
**C20:3 n6**	0	0.04	0.07	0.09	0.00	<0.01	<0.01	<0.01
	15	0.03	0.04	0.04	0.00			
**C20:4**	0	0.36	0.21	0.30	0.02	0.41	<0.01	<0.01
	15	0.11	0.21	0.14	0.02			
**C20:5**	0	0.05	0.13	0.40	0.02	<0.01	<0.01	<0.01
	15	0.04	0.13	0.10	0.03			
**C22:0**	0	0.05	0.05	0.05	0.00	<0.01	<0.01	<0.01
	15	0.04	0.08	0.06	0.01			
**C22:1**	0	0.01	0.02	0.04	0.00	<0.01	<0.01	<0.01
	15	0.04	0.02	0.04	0.01			
**C22:5**	0	0.19	0.30	0.56	0.02	<0.01	<0.01	<0.01
	15	0.14	0.18	0.23	0.02			
**C22:6**	0	0.09	0.21	0.54	0.02	<0.01	<0.01	<0.01
	15	0.05	0.13	0.19	0.02			

A quadratic effect was observed for C20:0 (*P *= 0.05, Table 6) and a marginal difference for C16:1C : 17 ante (*P *= 0.06), with increased concentrations in colostrum and milk in the 1% diet compared to the 0% and 2% diets. There was also a marginal quadratic difference for C17:1 (*P *= 0.08), where the 2% diet decreased the C17:1 concentration compared with the other two treatments. Additionally, there was a linear increase in C17iso (*P *= 0.04), C18:3 (*P *= 0.05), and C18:2t10c12 (*P *= 0.08) as the dietary EPA+DHA supplementation rate increased. On the other hand, in colostrum with increasing DHA+EPA in the diet a linear decrease in C18:0 and C18:1c12 (*P *≤ 0.03) and a marginal linear decreased in C18:1c9 (*P *≤ 0.09) was observed (Table 6). Consistent with our findings, previous studies have also reported an increased proportions of C18:3 in the colostrum of cows fed linseed compared to sunflower seed prepartum (Leiber et al., 2011). The fatty acid C18:2t10c12 is produced when biohydrogenation pathways are altered, and its concentration is associated with milk fat depression in ruminants (Bauman and Griinari, 2003). The C18:2t10c12 is important for its strong bioactive properties on lipid metabolism. Preclinical studies have demonstrated that C18:2t10c12 reduces body fat deposition by modulating adipocyte differentiation and lipid metabolism through pathways involving PPARγ (Kim et al., 2013). Feeding fatty acid sources like fish oil has been shown to increase concentrations of C18:2t10c12 in milk fat, potentially antagonizing the biohydrogenation of other fat sources (Palmquist and Griinari, 2006). Consequently, EPA and DHA supplementation has been linked to milk fat depression in both dairy cattle (Mattos et al., 2004; Shingfield et al., 2013) and sheep (Capper et al., 2007; Gallardo et al., 2014).

**Table 6.** Linear or quadratic effects on the fatty acid profile of colostrum (d 0) and milk at 15 d in lactation of increasing concentration of EPA and DHA supplementation (0%, 1%, 2% of calcium salts containing EPA and DHA) to ewes during the last 50 d of gestation (% of total fatty acid methyl esters).

	Treatment (Trt)				SEM	*P*-Value		
Fatty acid	Day	0%	1%	2%		Linear	Quadratic	Trt × day
**C17:0 iso**	0	0.41	0.49	0.52	0.04	0.04	0.57	0.81
	15	0.47	0.51	0.53	0.04			
**C16:1 17 ante**	0	2.18	2.50	2.34	0.10	0.28	0.06	0.80
	15	1.35	1.55	1.45	0.12			
**C17:1**	0	0.43	0.42	0.38	0.02	0.31	0.08	0.16
	15	0.36	0.42	0.34	0.03			
**C18:0**	0	5.54	4.17	3.74	0.40	0.01	0.89	0.17
	15	14.84	15.15	14.31	0.53			
**C18:1 c9**	0	24.92	21.73	19.69	1.42	0.09	0.97	0.28
	15	25.35	25.51	24.31	1.84			
**C18:1 c12**	0	0.27	0.15	0.11	0.04	0.03	0.07	0.23
	15	0.51	0.32	0.41	0.05			
**C18:3**	0	0.06	0.11	0.13	0.03	0.05	0.55	0.97
	15	0.13	0.19	0.20	0.04			
**C18:2 c12 t10**	0	0.02	0.03	0.03	0.00	0.08	0.46	0.12
	15	0.02	0.02	0.02	0.00			
**C20:0**	0	0.13	0.16	0.15	0.01	0.19	0.05	0.86
	15	0.19	0.23	0.21	0.01			

An interesting aspect of these results is that, despite the fact that dietary supplementation ending at parturition (d 0), elevated concentrations of fatty acids in milk were still observed 15 d postpartum. A similar pattern has been reported in humans where breast milk from women who received fish oil supplementation showed proportionally higher levels of DHA and EPA at 3 d and 6 wk after delivery, although this difference was no longer evident at 6 months (Dunstan et al., 2007). In contrast, studies in goats supplemented with a concentrate containing 9% PUFA-rich protected fat during the sixth month of lactation showed significant changes in their milk fatty acid profile (Sampelayo et al., 2004). Specifically, they had a reduction in saturated fatty acids and an increase in PUFA content, but these effects disappeared once the supplementation ceased (Sampelayo et al., 2004). These findings suggest that the timing of fatty acid supplementation may play a critical role in determining milk lipid composition, possibly through prepartum programming of the mammary gland, which induces long-lasting changes.

No differences were detected for other milk fatty acids (P ≥ 0.12; Supplementary Table 2). As expected, the fatty acids that showed no significant differences in milk and colostrum between supplemented and control ewes also presented no significant differences in other studies investigating the effects of PUFA supplementation on milk or colostrum fatty acids in sheep (Tsiplakou & Zervas, 2013; Coleman et al., 2018a)

### Lamb plasma

The primary fatty acids found in lamb plasma were C16:0, C18:0, C18:1t9, and C18:2 (Supplemental Table 3), which were also observed in ewe plasma in this experiment, as well as in previous studies (Coleman et al., 2018a). Garcia et al. (2014) showed that the fatty acid profile of offspring plasma can reflect dietary fatty acids given to dams during pregnancy (Garcia et al., 2014), as well as the fatty acids consumed through colostrum and milk (Capper et al., 2007; Or-Rashid et al., 2010). In the current experiment, lambs were blood sampled after colostrum intake, meaning the changes in their plasma fatty acid profile at birth reflect both ewe plasma and colostrum. Therefore, it’s unclear whether certain fatty acids were transferred through the placenta or consumed from the colostrum.

A quadratic effect was observed for C17iso, C18:1t9, C18:2, and C18:2c9t11 (*P *< 0.05; Table 7), with a marginal difference in the total n6 concentrations (*P *= 0.1), where the 1% supplementation group had greater concentrations than the 0% and 2% supplementation groups. The concentration of C20:5 showed a marginal quadratic effect (*P *= 0.09) with a lower concentration in the 1% supplemented group, while C14:1 exhibited a marginal quadratic effect (*P *= 0.06), with 0% and 1% supplemented diets having similar fatty acid concentration, while the 2% supplementation had the lowest concentration of C14:1. There was also an increasing linear effect for C18:1t11 and C22:6 (*P *≤ 0.01) and a marginal linear increase in DHA+EPA (P = 0.09), with concentrations rising as the dietary supplementation amount increased. Conversely, there was a linear decrease in C18:1c16 (*P *= 0.05) and a marginal decrease in both C18:1t6 (*P *= 0.08 and C18:1c12 (*P *= 0.06) as supplementation decreased. Previous studies reported increases in EPA and DHA in lamb plasma at birth when ewes were supplemented with EPA and DHA during pregnancy (Capper et al., 2006; Or-Rashid et al., 2010). Or-Rashid et al. (2010) also noted further increases in lamb plasma EPA and DHA after colostrum consumption in lambs born to fish meal–supplemented dams. However, Coleman et al. (2018) found that lamb plasma concentrations of EPA and DHA did not reflect the maternal or colostrum fatty acid profiles, possibly because circulating concentrations were insufficient to increase fetal transfer; their supplementation rate lower than the present experiment at 0.39% DM compared to our 1 and 2% of DM. No differences were detected for other fatty acids (P ≥ 0.12; Supplementary Table 3).

**Table 7.** Effect on lamb plasma fatty acids after colostrum consumption of increasing concentration of maternal EPA and DHA supplementation (0%, 1%, 2% of calcium salts containing EPA and DHA) during the last 50 d of gestation (% of total fatty acid methyl esters).

	Treatment			SEM	*P*-value	
	0%	1%	2%		Linear	Quadratic
**C 14:1**	0.21	0.28	0.07	0.06	0.10	0.06
**C 17:0 iso**	0.18	0.56	0.38	0.99	0.14	0.02
**C 18:1 t6**	0.72	0.58	0.44	0.12	0.08	0.98
**C 18:1 t9**	0.16	0.41	0.27	0.08	0.27	0.04
**C 18:1 t11**	0.47	1.63	1.74	0.35	0.01	0.12
**C 18:1 c12**	2.75	2.11	1.88	0.33	0.06	0.59
**C 18:1 c16**	0.04	0.00	0.00	0.02	0.05	0.25
**C 18:2**	2.49	4.42	3.07	0.73	0.55	0.05
**C 18:2 c9t11**	0.39	0.95	0.62	0.18	0.32	0.03
**C 20:5**	1.18	0.68	1.15	0.24	0.91	0.09
**C 22:6**	0.57	0.88	1.47	0.16	<0.01	0.47
**DHA + EPA**	1.78	1.56	2.62	0.35	0.09	0.14
**n6: n3**	0.90	1.28	0.85	0.20	0.85	0.10

The correlation analysis between colostrum and lamb plasma showed a positive correlation of short fatty acids, C10:0, C12:0, C13:0, C14:1, C15:0iso, C15:0ante, C15:0, C16:0, C17:0 iso, C17:1, C18:0, C18:1t6, C18:1t9, C18:2, C18:2c9t11, C20:1, C20:3n6, C20:3n3, C20:5, C22:1, C22:5, and C22:6 (*P *≤ 0.02; r ≥ 0.28; Table 8). These results show that the absorption of fatty acids in the lamb after birth is very high. However, the concentration of fatty acids in colostrum does not reflect the concentration of fatty acids in the plasma of the ewe. There was only a weak positive correlation between ewe plasma at lambing day and colostrum for C18:3 (*P *= 0.03; r = 0.30, Table 9). A greater concentration of C18:3 was found in the plasma of ewes, but not in the colostrum or in lambs, suggesting that C18:3 is not a preferentially selected fatty acid during colostrum formation. In contrast, C20:3n3 showed elevated concentrations in ewes plasma, colostrum, and lambs’ plasma, although ewe plasma had higher concentrations than lamb plasma. These findings suggest that there is no universal pattern in fatty acid selection during colostrum formation; rather, each fatty acid behaves differently in terms of transfer from the mother to the colostrum and from the mother to the lamb. Previous studies have shown that most n3LCFA present in maternal milk lipids originate from maternal body reserves rather than directly from the maternal diet (Larque et al., 2002). For C20:3n3 fatty acids specifically, it is understood that the mammary gland exhibits selective uptake, favoring the more biologically efficient or preferred form when multiple types are available in circulation (Fidler et al., 2000; Innis, 2007).

**Table 8:** Correlation between fatty acid concentration of colostrum and lamb plasma when dams were supplemented with increasing concentrations EPA and DHA (0%, 1%, 2% of calcium salts containing EPA and DHA) during the last 50 d of gestation.

Fatty acid	r	*P*-value
**Short FA**	0.35	0.01
**C10:0**	0.44	<0.01
**C12:0**	0.49	<0.01
**C13:0**	0.7	<0.01
**C14:0 iso**	0.19	0.13
**C14:0**	−0.05	0.66
**C14:1**	0.38	<0.01
**C15:0 iso**	0.47	<0.01
**C15:0 ante**	0.59	<0.01
**C15:0**	0.48	<0.01
**C16:0 iso**	0.19	0.13
**C16:0**	0.35	<0.01
**C17:0 iso**	0.54	<0.01
**C16:1 c17 ante **	0.10	0.40
**C17:1**	0.29	0.02
**C18:0**	0.32	0.01
**C18:1t6**	0.36	0.01
**C18:1t9**	0.55	<0.01
**C18:1c9**	0.54	<0.01
**C18:2**	0.28	0.02
**C18:3**	0.14	0.26
**C18:2 c9t11**	0.39	<0.01
**C20:0**	0.12	0.31
**C20:1**	0.36	<0.01
**C20:3 n6**	0.31	0.01
**C20:3 n3**	0.36	<0.01
**C20:4**	0.12	0.34
**C20:5**	0.47	<0.01
**C22:0**	0.50	<0.01
**C22:1**	0.36	<0.01
**C22:5**	0.47	<0.01
**C22:6**	0.53	<0.01

**Table 9:** Correlation between fatty acid concentration of colostrum and ewe plasma at lambing when the ewes were supplemented with increasing concentrations EPA and DHA (0%, 1%, 2% of calcium salts containing EPA and DHA) during the last 50 d of gestation.

Fatty acid	r	*P*-value
**Short FA**	0.05	0.67
**C14:0**	−0.19	0.14
**C14:1**	−0.17	0.15
**C15:0 iso**	0.02	0.87
**C15:0 ante**	−0.07	0.56
**C15:0**	−0.05	0.72
**C16:0 iso**	0.17	0.20
**C16:0**	0.08	0.50
**C17:0iso**	−0.11	0.36
**C16:1 c17ante **	0.03	0.78
**C17:1**	0.18	0.17
**C18:0**	−0.17	0.18
**C18:1t6**	−0.02	0.85
**C18:1t9**	0.04	0.78
**C18:1c9**	0.18	0.26
**C18:2**	0.06	0.65
**C18:3**	0.30	0.03
**C18:2c9t11**	0.00	0.99
**C20:0**	0.02	0.72
**C20:1**	−0.03	0.71
**C20:3 n6**	0.00	0.94
**C20:3 n3**	−0.00	0.97
**C20:4**	0.09	0.47
**C20:5**	0.10	0.43
**C22:0**	−0.12	0.40
**C22:1**	−0.11	0.42
**C 22:5**	−0.17	0.21
**C 22:6**	0.06	0.67

## Conclusion

Supplementation with EPA and DHA modified fatty acid concentration in ewe plasma, colostrum and lamb plasma. Although, EPA and DHA supplementation increases the concentration of n3LCFA in the plasma of the ewe, this increase in plasma n3LCFA was not reflected in a positive correlation with the increase in colostrum at parturition, colostrum did present a greater concentration of n3LCFA. Due to the high intestinal absorption of the lamb at birth, these polyunsaturated fatty acids also increase their concentration in the plasma of lambs. This increase may play an important role in the lipid metabolism of lambs.

## Supplementary Material

skaf366_Supplementary_Data

## Abbreviations

BCS: body condition score
DHA: docosahexaenoic acid
EPA: eicosapentaenoic acid
FA: fatty acid
n-3: omega-3
n3LCFA: omega-3 long chain fatty acids
PUFA: polyunsaturated fatty acids
