# Supplementary material for: Supplementation with eicosapentaenoic and docosahexaenoic acids during late gestation alters fatty acid profiles in ewe colostrum, milk, and plasma, and lamb plasma
Source: J Anim Sci. 2025 Nov 16;103:skaf366. doi: 10.1093/jas/skaf366 (PMC12619979; doi:10.1093/jas/skaf366)
Supplement: skaf366_Supplementary_Data [file skaf366_supplementary_data.zip › Supplementary Table 1 S104 JAS.docx]

| **Supplementary Table 1**. Effects on plasma fatty acid profile of increasing concentration of EPA and DHA supplementation (0%, 1%, 2% of calcium salts containing EPA and DHA) to ewes during the last 50 d of gestation. Samples were taken d -20 before parturition, the day of lambing (d 0), and d 15 after parturition (% of total fatty acid methyl esters). | | | | | | | | |
| --- | --- | --- | --- | --- | --- | --- | --- | --- |
|  |  | Treatment (Trt) | | | SEM | P-Values | | |
| Fatty acid | Day | 0% | 1% | 2% |  | Linear | Quadratic | Trt x day |
| Short FA | -20 | 0.01 | 0.00 | 0.00 | 2.86 | 0.82 | 0.80 | 0.14 |
|  | 0 | 14.99 | 20.84 | 12.59 | 3.28 |  |  |  |
|  | 15 | 11.27 | 7.94 | 15.42 | 3.01 |  |  |  |
| C13:0 | -20 | 0.00 | 0.00 | 0.00 | 0.02 | 0.26 | 0.52 | 0.60 |
|  | 0 | 0.00 | 0.06 | 0.01 | 0.02 |  |  |  |
|  | 15 | 0.01 | 0.00 | 0.05 | 0.02 |  |  |  |
| C14:0 | -20 | 0.06 | 0.27 | 0.24 | 0.11 | 0.74 | 0.53 | 0.19 |
|  | 0 | 0.61 | 0.57 | 0.57 | 0.12 |  |  |  |
|  | 15 | 0.89 | 0.60 | 0.85 | 0.11 |  |  |  |
| C15iso | -20 | 0.04 | 0.11 | 0.09 | 0.04 | 0.68 | 0.86 | 0.31 |
|  | 0 | 0.11 | 0.04 | 0.03 | 0.05 |  |  |  |
|  | 15 | 0.13 | 0.16 | 0.16 | 0.04 |  |  |  |
| C14:1 | -20 | 0.59 | 0.40 | 0.58 | 0.08 | 0.98 | 0.95 | 0.5 |
|  | 0 | 0.00 | 0.01 | 0.06 | 0.09 |  |  |  |
|  | 15 | 0.05 | 0.23 | 0.01 | 0.08 |  |  |  |
| C15:0 | -20 | 0.70 | 0.86 | 0.80 | 0.10 | 0.79 | 0.97 | 0.75 |
|  | 0 | 0.41 | 0.34 | 0.40 | 0.11 |  |  |  |
|  | 15 | 0.46 | 0.42 | 0.45 | 0.10 |  |  |  |
| C16:0 iso | -20 | 0.55 | 0.38 | 0.32 | 0.06 | 0.60 | 0.45 | 0.64 |
|  | 0 | 0.24 | 0.09 | 0.10 | 0.07 |  |  |  |
|  | 15 | 0.23 | 0.39 | 0.30 | 0.06 |  |  |  |
| C17:0 iso | -20 | 0.75 | 0.93 | 0.12 | 0.09 | 0.48 | 0.88 | 0.24 |
|  | 0 | 0.51 | 0.46 | 0.47 | 0.10 |  |  |  |
|  | 15 | 0.39 | 0.51 | 0.35 | 0.09 |  |  |  |
| C17:0 | -20 | 1.45 | 1.54 | 1.61 | 0.13 | 0.61 | 0.81 | 0.64 |
|  | 0 | 1.29 | 1.18 | 1.19 | 0.15 |  |  |  |
|  | 15 | 1.30 | 1.14 | 1.05 | 0.14 |  |  |  |
| C18:1t9 | -20 | 0.17 | 0.38 | 0.67 | 0.15 | 0.14 | 0.93 | 0.47 |
|  | 0 | 0.09 | 0.13 | 0.12 | 0.17 |  |  |  |
|  | 15 | 0.08 | 0.10 | 0.16 | 0.15 |  |  |  |
| C18:1t12 | -20 | 1.06 | 0.89 | 0.68 | 0.22 | 0.55 | 0.92 | 0.86 |
|  | 0 | 0.31 | 0.42 | 0.40 | 0.25 |  |  |  |
|  | 15 | 0.40 | 0.33 | 0.32 | 0.23 |  |  |  |
| C18:1c11 | -20 | 1.03 | 1.07 | 1.45 | 0.31 | 0.37 | 0.37 | 0.16 |
|  | 0 | 0.28 | 0.23 | 0.19 | 0.35 |  |  |  |
|  | 15 | 1.30 | 0.32 | 0.24 | 0.32 |  |  |  |
| C18:1c12 | -20 | 0.68 | 0.58 | 0.62 | 0.10 | 0.68 | 0.71 | 0.87 |
|  | 0 | 0.64 | 0.52 | 0.62 | 0.12 |  |  |  |
|  | 15 | 0.61 | 0.68 | 0.58 | 0.11 |  |  |  |
| C18:1c16 | -20 | 0.04 | 0.02 | 0.00 | 0.02 | 0.13 | 0.84 | 0.91 |
|  | 0 | 0.03 | 0.03 | 0.02 | 0.02 |  |  |  |
|  | 15 | 0.02 | 0.00 | 0.00 | 0.02 |  |  |  |
| C18:2 | -20 | 0.00 | 0.05 | 0.03 | 0.04 | 0.66 | 0.46 | 0.65 |
|  | 0 | 0.10 | 0.21 | 0.12 | 0.05 |  |  |  |
|  | 15 | 0.06 | 0.17 | 0.04 | 0.04 |  |  |  |
| C20:0 | -20 | 21.64 | 21.95 | 20.98 | 1.10 | 0.66 | 0.50 | 0.64 |
|  | 0 | 15.81 | 14.49 | 14.83 | 1.26 |  |  |  |
|  | 15 | 16.34 | 18.47 | 16.62 | 1.15 |  |  |  |
| C20:1 | -20 | 0.00 | 0.00 | 0.00 | 0.16 | 0.58 | 0.94 | 0.31 |
|  | 0 | 0.36 | 0.09 | 0.28 | 0.19 |  |  |  |
|  | 15 | 1.37 | 1.81 | 1.73 | 0.18 |  |  |  |
| C18:3 | -20 | 1.78 | 1.63 | 1.52 | 0.18 | 0.42 | 0.33 | 0.88 |
|  | 0 | 1.01 | 0.70 | 0.83 | 0.21 |  |  |  |
|  | 15 | 1.09 | 0.96 | 1.14 | 0.19 |  |  |  |
| C21:0 | -20 | 0.00 | 0.09 | 0.03 | 1.69 | 0.92 | 0.80 | 0.99 |
|  | 0 | 4.94 | 5.63 | 4.38 | 1.92 |  |  |  |
|  | 15 | 0.00 | 0.00 | 0.00 | 1.76 |  |  |  |
| C20:3n3 | -20 | 0.13 | 0.19 | 0.02 | 0.17 | 0.58 | 0.76 | 0.97 |
|  | 0 | 0.00 | 0.00 | 0.03 | 0.20 |  |  |  |
|  | 15 | 1.09 | 1.02 | 0.91 | 0.18 |  |  |  |
| C24:0 | -20 | 0.16 | 0.00 | 0.18 | 0.08 | 0.62 | 0.21 | 0.82 |
|  | 0 | 0.03 | 0.02 | 0.05 | 0.09 |  |  |  |
|  | 15 | 0.06 | 0.07 | 0.11 | 0.08 |  |  |  |
| n6 | -20 | 22.14 | 22.98 | 21.83 | 1.17 | 0.64 | 0.34 | 0.55 |
|  | 0 | 18.21 | 15.72 | 16.18 | 1.35 |  |  |  |
|  | 15 | 17.95 | 19.31 | 17.22 | 1.24 |  |  |  |
