# Supplementary material for: Supplementation with eicosapentaenoic and docosahexaenoic acids during late gestation alters fatty acid profiles in ewe colostrum, milk, and plasma, and lamb plasma
Source: J Anim Sci. 2025 Nov 16;103:skaf366. doi: 10.1093/jas/skaf366 (PMC12619979; doi:10.1093/jas/skaf366)
Supplement: skaf366_Supplementary_Data [file skaf366_supplementary_data.zip › Supplementary Table 2 S104 JAS.docx]

**Supplementary Table 2**. Effects on the fatty acid profile of colostrum (d 0) and milk at 15 d in lactation from ewes under increasing concentration of EPA and DHA supplementation (0%, 1%, 2% of calcium salts containing EPA and DHA) during the last 50 d of gestation (% of total fatty acid methyl esters).

|  |  | Treatment (Trt) | | | SEM | P-Values | | |
| --- | --- | --- | --- | --- | --- | --- | --- | --- |
| Fatty acid | Day | 0% | 1% | 2% |  | Linear | Quadratic | Trt x day |
| Short FA | 0 | 10.08 | 10.009 | 10.48 | 0.69 | 0.65 | 0.61 | 0.99 |
|  | 15 | 13.83 | 13.60 | 14.11 | 0.77 |  |  |  |
| C8:0 | 0 | 0.89 | 0.87 | 0.95 | 0.07 | 0.20 | 0.42 | 0.86 |
|  | 15 | 1.60 | 1.61 | 1.74 | 0.09 |  |  |  |
| C10:0 | 0 | 2.43 | 2.42 | 2.61 | 0.23 | 0.56 | 0.50 | 0.96 |
|  | 15 | 4.46 | 4.31 | 4.59 | 0.30 |  |  |  |
| C12:0 | 0 | 2.73 | 2.70 | 2.84 | 0.17 | 0.83 | 0.60 | 0.93 |
|  | 15 | 2.58 | 2.47 | 2.56 | 0.21 |  |  |  |
| C13:0 ante | 0 | 0.04 | 0.04 | 0.04 | 0.01 | 0.51 | 0.29 | 0.42 |
|  | 15 | 0.01 | 0.00 | 0.02 | 0.01 |  |  |  |
| C13:0 | 0 | 0.06 | 0.05 | 0.06 | 0.00 | 0.48 | 0.14 | 0.73 |
|  | 15 | 0.06 | 0.06 | 0.06 | 0.01 |  |  |  |
| C14 iso | 0 | 0.06 | 0.05 | 0.05 | 0.01 | 0.25 | 0.94 | 0.39 |
|  | 15 | 0.11 | 0.11 | 0.11 | 0.01 |  |  |  |
| C14:0 | 0 | 13.60 | 14.12 | 14.05 | 0.62 | 0.87 | 0.79 | 0.61 |
|  | 15 | 6.66 | 5.92 | 4.46 | 0.82 |  |  |  |
| C14:1 | 0 | 0.60 | 0.66 | 0.54 | 0.05 | 0.63 | 0.37 | 0.47 |
|  | 15 | 0.07 | 0.07 | 0.07 | 0.06 |  |  |  |
| C15 iso | 0 | 0.17 | 0.17 | 0.18 | 0.01 | 0.17 | 0.61 | 0.67 |
|  | 15 | 0.26 | 0.28 | 0.28 | 0.01 |  |  |  |
| C15 ante | 0 | 0.22 | 0.20 | 0.21 | 0.01 | 0.26 | 0.71 | 0.22 |
|  | 15 | 0.34 | 0.37 | 0.38 | 0.02 |  |  |  |
| C15:0 | 0 | 0.64 | 0.58 | 0.63 | 0.04 | 0.59 | 0.30 | 0.67 |
|  | 15 | 0.73 | 0.73 | 0.80 | 0.05 |  |  |  |
| C16:0 | 0 | 29.33 | 30.90 | 30.09 | 0.96 | 0.64 | 0.85 | 0.40 |
|  | 15 | 22.32 | 21.73 | 22.72 | 1.21 |  |  |  |
| C18:1t9 | 0 | 0.34 | 0.43 | 0.36 | 0.06 | 0.12 | 0.34 | 0.36 |
|  | 15 | 0.34 | 0.49 | 0.55 | 0.08 |  |  |  |
| C18:1 t12 | 0 | 0.30 | 0.50 | 0.49 | 0.25 | 0.87 | 0.25 | 0.41 |
|  | 15 | 0.84 | 1.34 | 0.74 | 0.29 |  |  |  |
| C20:1 | 0 | 0.46 | 0.42 | 0.48 | 0.04 | 0.93 | 0.63 | 0.25 |
|  | 15 | 0.46 | 0.53 | 0.45 | 0.05 |  |  |  |
| C20:3 n3 | 0 | 0.01 | 0.01 | 0.01 | 0.00 | 0.24 | 0.56 | 0.23 |
|  | 15 | 0.00 | 0.00 | 0.00 | 0.00 |  |  |  |
