# Supplementary material for: Supplementation with eicosapentaenoic and docosahexaenoic acids during late gestation alters fatty acid profiles in ewe colostrum, milk, and plasma, and lamb plasma
Source: J Anim Sci. 2025 Nov 16;103:skaf366. doi: 10.1093/jas/skaf366 (PMC12619979; doi:10.1093/jas/skaf366)
Supplement: skaf366_Supplementary_Data [file skaf366_supplementary_data.zip › Supplementary Table 3 s104 jas.docx]

**Supplementary Table 3.** Effect on lamb plasma fatty acids after colostrum consumption of increasing concentration of maternal EPA and DHA supplementation (0%, 1%, 2% of calcium salts containing EPA and DHA) during the last 50 d of gestation (% of total fatty acid methyl esters).

|  | Treatment | | | SEM | P-values | |
| --- | --- | --- | --- | --- | --- | --- |
|  | 0% | 1% | 2% |  | Linear | Quadratic |
| Short FA | 0.62 | 1.16 | 1.36 | 0.42 | 0.19 | 0.73 |
| C10:0 | 0.05 | 0.20 | 0.31 | 0.14 | 0.16 | 0.89 |
| sC12:0 | 0.57 | 0.95 | 1.04 | 0.30 | 0.24 | 0.67 |
| C13:0 | 0.22 | 0.00 | 0.01 | 0.12 | 0.21 | 0.44 |
| C14 iso | 0.01 | 0.00 | 0.01 | 0.01 | 0.71 | 0.24 |
| C14:0 | 4.65 | 4.77 | 4.17 | 0.94 | 0.70 | 0.75 |
| C15:0 iso | 0.20 | 0.24 | 0.10 | 0.07 | 0.27 | 0.29 |
| C15:0 ante | 0.07 | 0.19 | 0.13 | 0.06 | 0.48 | 0.21 |
| C15:0 | 0.58 | 0.70 | 0.73 | 0.08 | 0.16 | 0.64 |
| C16 iso | 0.05 | 0.07 | 0.03 | 0.03 | 0.68 | 0.39 |
| C16:0 | 29.70 | 29.64 | 30.91 | 1.13 | 0.43 | 0.62 |
| C16:1 c17 ante | 2.49 | 2.21 | 2.25 | 0.25 | 0.47 | 0.60 |
| C17:1 | 0.39 | 0.61 | 0.49 | 0.09 | 0.40 | 0.12 |
| C18:0 | 12.41 | 11.52 | 11.89 | 0.79 | 0.62 | 0.49 |
| C18:1 t10 | 0.68 | 0.13 | 0.96 | 0.23 | 0.36 | 0.25 |
| C18:1 t12 | 0.99 | 0.88 | 0.86 | 0.17 | 0.58 | 0.84 |
| C18:1 c9 | 27.73 | 25.15 | 25.92 | 1.63 | 0.41 | 0.39 |
| C18:1 c11 | 2.80 | 2.84 | 2.75 | 0.33 | 0.90 | 0.86 |
| C18:1 c13 | 0.07 | 0.07 | 0.01 | 0.05 | 0.34 | 0.70 |
| C18:1 c15 | 0.01 | 0.00 | 0.08 | 0.05 | 0.31 | 0.41 |
| C18:3 | 0.37 | 0.51 | 0.16 | 0.17 | 0.38 | 0.23 |
| C20:0 | 0.00 | 0.05 | 0.01 | 0.03 | 0.80 | 0.25 |
| C20:1 | 0.00 | 0.00 | 0.07 | 0.03 | 0.16 | 0.36 |
| C20:3 n6 | 0.00 | 0.23 | 0.00 | 0.14 | 1.00 | 0.16 |
| C20:3 n3 | 0.00 | 0.07 | 0.02 | 0.04 | 0.77 | 0.14 |
| C20:4 | 1.64 | 1.84 | 1.75 | 0.21 | 0.69 | 0.55 |
| C22:0 | 1.43 | 1.23 | 1.09 | 0.25 | 0.31 | 0.88 |
| C22:1 | 0.71 | 0.16 | 0.39 | 0.21 | 0.27 | 0.14 |
| C24:0 | 0.06 | 0.08 | 0.07 | 0.07 | 0.86 | 0.88 |
| C22:5 | 1.12 | 0.68 | 0.94 | 0.20 | 0.53 | 0.14 |
